# The Parkinson's Puzzle Box

**DOI:** 10.1111/hex.14116

**Published:** 2024-06-19

**Authors:** Shafaq Hussain‐Al, Jane Alty, Michele Callisaya

**Affiliations:** ^1^ Yorkshire UK; ^2^ Wicking Dementia Research and Education Centre University of Tasmania Hobart Tasmania Australia; ^3^ School of Medicine University of Tasmania Hobart Tasmania Australia; ^4^ Neurology Department Royal Hobart Hospital Hobart Tasmania Australia; ^5^ Menzies Institute for Medical Research University of Tasmania Hobart Tasmania Australia; ^6^ Peninsula Clinical School Monash University Frankston Victoria Australia; ^7^ National Centre for Healthy Ageing Monash University and Peninsula Health Frankston Australia

**Keywords:** diagnosis, early onset, gender, health, Parkinson's disease, person‐centred care, women, younger onset

## Abstract

**Introduction:**

Women and those with younger onset Parkinson's Disease (YOPD) are typically diagnosed later and face unique situations and challenges. This essay aims to raise awareness of the difficulties in diagnosing YOPD and the need for a personalised approach to care for women with YOPD.

**Methods:**

Two professional women with YOPD (academic physiotherapist and practicing dentist) and a female neurologist (clinician academic) came together to write a narrative essay on their personal experience and perspectives in relation to women and YOPD.

**Results:**

The essay outlines how the experience of diagnosis is likened to a complex puzzle box with many interlocking components that are hidden and difficult to solve. The concerns of the women about their identity, work, family and the future, with most supports targeting those that are older and retired are outlined.

**Conclusion:**

It is concluded that YOPD is a complex puzzle to solve, but can be done by understanding all the intricate interlocking components of the puzzle and combined with greater awareness could lead to earlier diagnosis and the delivery of successful person‐centred care.

**Patient or Public Contribution:**

People with lived experience were involved in the essay conception and writing.

## 1 Introduction

Have you ever seen a puzzle box? It is a seemingly simple wooden box that fits in the palm of your hand, but inside it contains an intricate pattern of interlocking components. The puzzle is to work out how those components piece together, so you can open it up to reveal the secrets within. There will be slots, catches and notches, all of which will need to be ordered precisely to create the perfect alignment before the puzzle is solved. In some ways, the human body is similar, with multiple parts that need to be synchronised and ordered to allow it to properly function. With this essay, we aim to challenge the way problems are perceived, encouraging the reader to think about how apparently simple symptoms sometimes signal unexpected hidden complexities and the need to think about new ways to solve the deceptively simple ‘puzzle box’.

## 2 The Diagnostic Puzzle

So now let your mind wander to imagine yourself sitting in a clinic room, consulting with a white 49‐year‐old physiotherapist who works in a local public hospital and lives with her partner of 12 years. She is a keen mountain runner, who looks fit and healthy but describes 6 months of muscle cramps in her toes and calf after running for about 20 min. Symptoms disappear immediately on stopping and 4 weeks of physiotherapy has not helped. She also reports tingling in her lower legs and a feeling of tiredness and low mood. All these symptoms started a few months after bilateral oophorectomy (no cancer found) and she has just started hormone replacement therapy. She reports a maternal family history of breast cancer, but nothing further of note. What diagnosis springs to mind? Could it be a sporting injury, or perhaps a lumbar nerve root compression or is it all due to anxiety? We'll leave you to ponder upon this a while…

In the meantime, a second patient presents; this time a 39‐year‐old dentist of South Asian descent with a strong family history of cardiovascular disease and type II diabetes. She works in a busy dental surgery and is married with two young children. She presents with extreme fatigue and a vague pain, sometimes with pins and needles sensations, in her hands and feet. During the consult, she is teary, and not sure why, but manages to explain that she constantly feels she is juggling too many balls. She often feels anxious and likens this feeling to ‘not being able to catch one's breath’.

What you conclude about the likely diagnosis in each of these cases will, of course, be coloured by what type of clinic you are running. If I told you both women had the same condition, you might wonder if the unifying diagnosis could be menopause or perhaps depression or a vitamin deficiency. All reasonable differentials and those were the conditions tested for first in each case. But what if I said both women have young onset Parkinson's disease (YOPD), where the symptoms develop in those under 50 years? Would you have been able to piece together the symptoms to unlock the puzzle box? Would you have known that these vague symptoms are how Parkinson's can start? We know this because this is how it started in us—the physiotherapist and the dentist. Subtle and episodic, in fit young women who know how to keep going, so competent at compensating.

Back to the physiotherapist—where the doctor didn't yet have all the pieces to the diagnostic puzzle. Over time, the physiotherapist developed a sore neck and difficulty typing. Running stopped and work became harder. She visited other doctors and had scans of her spine and brain, but no one knew how to solve the puzzle box. Finally, a tremor and lack of arm swing on the left side brought her back to the original doctor. Two years after those initial symptoms, the intricate interlocking components came together to spring open the puzzle box—a diagnosis of Parkinson's disease.

And back to the dentist. What started off as vague pain, fatigue and anxiety—isolated spots of concern—grew and coalesced until it became obvious that these weren't isolated symptoms. They are all interconnected, and only by examining them as a whole were the components of the box joined together to reach the correct conclusion. The final two parts of the puzzle that solved the mystery were the tremor becoming more obvious, the right arm truly losing its swing, with the dentist cradling her right arm—holding it almost like a broken wing. As these two pieces fell into place, it became obvious that YOPD was the likely diagnosis.

**Figure 1:**
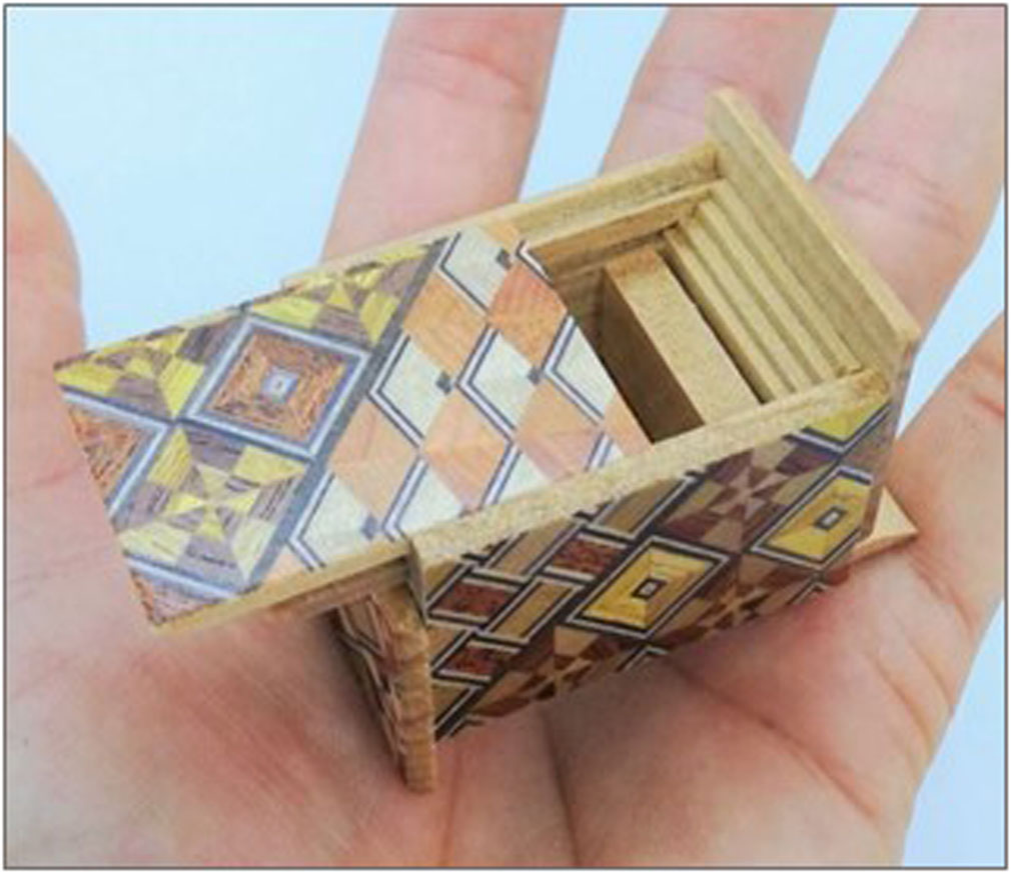
Just like the simplicity of the wooden box that eludes us to the complexities that lie within, youth, femininity and fitness blind our perception to see the subtle early symptoms and signs of what is peeking back at us.

And yet capable young women are a far cry from how Parkinson's was taught to us in university when we were shown ‘that’ famous drawing of the man with Parkinson's—so old, so hunched, so male. A far cry too from the famous people who have declared their diagnoses on our TV screens in recent years—Michael J. Fox, Pope John‐Paul II and Billy Connolly. Images are powerful and they shape what we see—they are the instructions to help us solve the puzzle. Except the wrong instructions have been put with the puzzle; no shakes nor shuffling but still Parkinson's peeks at us. Just like the simplicity of the wooden box that eludes us to the complexities that lie within, youth and femininity and fitness blind our perception so we can't see the subtle early symptoms and signs (Figure 1). We don't look for Parkinson's in the young, and especially not in young women.

## 3 The Puzzle of Supporting Women With YOPD

Why does being young and female matter? Well, they go undiagnosed for longer, receive medication later, develop more anxiety and ‘see’ into their future longer without any clear direction [1]. Furthermore, women and those from ethnic backgrounds have less access to quality care [2].

Although the diagnostic puzzle is ‘solved’—the box then opens a journey that can prove different from those who are older. Those diagnosed when younger have worse psychological wellbeing, greater perceived stigma and face a long road of uncertainty [3, 4]. Uncertainty about work, uncertainty about their ability to look after children, uncertainty about relationships and uncertainty around their ability to afford medications or brain surgery. Medical management is tailored to symptoms in the clinic rather than for chasing a child, practising dentistry or running the trails. Support groups largely target retired older people, leaving those with younger onset feeling isolated and alone.

And here lies the crux for us neurologists—we were not taught how to recognise, or how to treat, YOPD—we learn it from our patients and from our mistakes. And as the incidence of Parkinson's rises around the world, amongst people of all ages, our ability to diagnose needs to become sharper and more personalised. Those diagnosed with YOPD are too often left to solve their own puzzle. They learn from trial and error, or from others going through the same experience connecting via social media platforms.

That is how we three women now learn from each other—the physiotherapist in Australia and the dentist in England, connected by a mutual friend, the neurologist. We are re‐drawing the image of Parkinson's so that we can remind others to solve the puzzle box as a whole. Rather than just focusing on a bit of the puzzle—the older, white male—we want to look at all the cogs, regardless of age or gender, and we want the world to step back and take a wider look at how we perceive Parkinson's disease. As health professionals, we wish our own professions learnt more about the ‘slots, catches and notches’ of the younger Parkinson's puzzle. As younger women living with Parkinson's, we are raising our voices through media and advocacy, so that the community know about younger women and Parkinson's, and so others travelling the same journey don't feel so alone. We hope that doctors ask us about work, sport, relationships and family, that medications are tailored to these activities and that referrals are made to allied health professionals with Parkinson's knowledge to support us. We may want to meet others of a similar age—please help us connect so we feel less isolated and can learn from, and help, others who are walking this path. Help us educate family, friends and workplaces on both motor and nonmotor symptoms, delivered in ways that don't add to the stigma, uncertainty and fear already experienced.

As a neurologist, I have learned far more about the intricacies of YOPD in women, from my two friends (co‐authors) than anything I gleaned in medical school, trudging the wards or even my doctorate degree in Parkinson's. Together with the young‐onset patients whom I have walked alongside over the years, on their own personal journeys, I have come to realise that we desperately need new ways to solve the Parkinson's puzzle to deliver personalised care, especially so for young women.

In women, it is clear that the components and experiences that make up the puzzle of living with Parkinson's are different from many men. But there is still so much to learn, with an underrepresentation of women in Parkinson's clinical trials, and few papers focusing on these issues in women [5]. What is increasingly clear is that many people with Parkinson's, regardless of gender, lack information on their condition and how lifestyle interventions and supports can help maintain participation in life's various roles. This sense of ‘being stuck in the dark’ leads to increased uncertainty, anxiety and lost autonomy. Thus, a cornerstone of good, personalised care for Parkinson's starts with providing access to timely education and allowing the patient to participate in their own health decisions. Information and discussions should be tailored to the individual's features such as age, gender, racial and cultural background, as well as employment, family roles and leisure activities. Furthermore, the current method of delivering nearly all care through hospital clinics during working hours tends to be particularly disruptive for women with YOPD who are more likely to be in employment or have dependent children at home. A solution could be to harness the ubiquity of laptops or mobile devices to deliver education, monitoring and care into the home or office.

As a group of healthcare professionals and women with lived experience of Parkinson's, we wonder if we have appreciated all the idiosyncrasies that make the puzzle box. Whether we have appreciated how each puzzle box can look so similar, and yet be so intrinsically different. We ask you to think about us when you are out running, sitting in the dentist's chair or sitting opposite that young woman in your clinic. Come run with us on this path, and open your mind to *all* the intricacies of each individual with Parkinson's. Open your minds to thinking outside, and within, the box so that we may better understand the puzzle that presents as Parkinson's disease. So that we may indeed, one day, solve the puzzle that is Parkinson's disease.

### 3.1 Postscript

Parkinson's disease is the second most common neurodegenerative condition after Alzheimer's disease. Globally, the prevalence is over 8.5 million, with a higher prevalence in men than women (1.4:1). Parkinson's disease is characterised by both motor (bradykinesia, rigidity, postural instability, tremor) and nonmotor symptoms (e.g., depression, anxiety, cognitive impairment, sleep disorders, constipation, postural hypotension). The diagnosis is based on clinical evaluation of symptoms and signs and can often take years after initial symptoms appear. Perceptions of Parkinson's disease are mostly of a stooped old white man with a tremor and shuffling gait. Differences between men and women in symptoms in the prodromal and clinical stages may lead to a longer time to diagnosis for women [5], and few realise that approximately 20% of people are of working age and 10% are 50 years or under. Diagnosis at a younger age and being female can lead to unique challenges with work, relationships and family responsibilities, changes in symptoms with pregnancy and menopause and higher rates of osteoporosis increase the risk of fractures [5]. There have been recent calls for greater research and frameworks to improve the understanding, diagnosis and care of women with Parkinson's disease [5]. We hope that this narrative essay contributes to this call for action.

## Author Contributions

**Shafaq Hussain‐Al:** conceptualisation, methodology, writing–original draft, writing–review and editing. **Jane Alty:** conceptualisation, methodology, writing–original draft, writing–review and editing. **Michele Callisaya:** conceptualisation, methodology, writing–ordinal draft, writing–review and editing.

## Conflicts of Interest

The authors declare no conflicts of interest.

## Data Availability

Data sharing is not applicable to this article as no new data were created or analysed in this study.
